# Automated high-content image-based characterization of microorganism behavioral diversity and distribution

**DOI:** 10.1016/j.csbj.2023.10.055

**Published:** 2023-11-02

**Authors:** Carlotta Aurora Lupatelli, Agnes Attard, Marie-Line Kuhn, Celine Cohen, Philippe Thomen, Xavier Noblin, Eric Galiana

**Affiliations:** a Université Côte d’Azur, INRAE, CNRS, ISA, Sophia Antipolis, 06903, France; b Université Côte d’Azur, CNRS, UMR 7010, INPHYNI, Nice 06200, France

**Keywords:** Microorganisms, Micro-environments, Drivers, Distribution, Automated image analysis, Cell tracking

## Abstract

Microorganisms have evolved complex systems to respond to environmental signals. Gradients of particular molecules and elemental ions alter the behavior of microbes and their distribution within their environment. Microdevices coupled with automated image-based methods are now employed to analyze the instantaneous distribution and motion behaviors of microbial species in controlled environments at small temporal scales, mimicking, to some extent, macro conditions. Such technologies have so far been adopted for investigations mainly on individual species. Similar versatile approaches must now be developed for the characterization of multiple and complex interactions between a microbial community and its environment. Here, we provide a comprehensive step-by-step method for the characterization of species-specific behavior in a synthetic mixed microbial suspension in response to an environmental driver. By coupling accessible microfluidic devices with automated image analysis approaches, we evaluated the behavioral response of three morphologically different telluric species (*Phytophthora parasitica*, *Vorticella microstoma*, *Enterobacter aerogenes*) to a potassium gradient driver. Using the TrackMate plug-in algorithm, we performed morphometric and then motion analyses to characterize the response of each microbial species to the driver. Such an approach enabled to confirm the different morphological features of the three species and simultaneously characterize their specific motion in reaction to the driver and their co-interaction dynamics. By increasing the complexity of suspensions, this approach could be integrated in a framework for phenotypic analysis in microbial ecology research, helping to characterize how key drivers influence microbiota assembly at microbiota host-environment interfaces.

## Graphical Abstract

**Figure:**
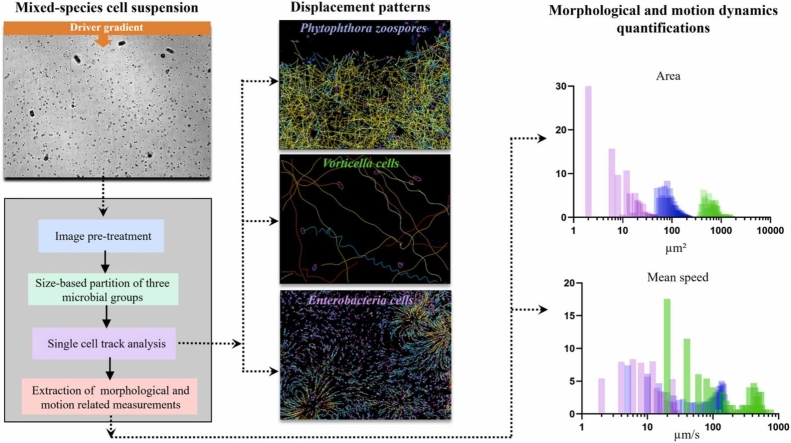

## 1 Introduction

Microbiota are shaped primarily by environmental drivers. In ground ecosystems, soil parameters (i.e., pH, soil texture, mineral fluxes), biotic interactions and soil management appear to be the main drivers of the composition and dynamics of telluric microbiomes [1]. Depending on soil properties and uses, microbial dynamics may vary significantly on large time scales (years, months, and days). To explore which drivers affect soil microbial dynamics at these large time scales, and how they do so, different omics approaches have been developed [2], [3], [4], [5]. For example, high-throughput barcoded pyrosequencing of the 16S rRNA gene was used to compare taxa diversity and differences in community compositions within and between land-use types, revealing substantial monthly variability [6]. Effects of drivers on microbial diversity occurring at shorter time scales (days, hours) have also been studied. Computational simulations coupled with experimental microjunction surface sampling probe mass spectrometry applied on a specially designed rhizosphere-on-a-chip, enabled the detection of root-exuded hotspots over a period of 2 h to 12 days that potentially impact the rhizospheric microbiota distribution [7]. Drivers acting on time scales of seconds to minutes also play key roles in microbiota assembly and their characterization is required. For example, they may determine heterogeneity in spatial dispersions of species, as in latent stage versus spore germination, or in the displacement and guidance of swimming cells [8], [9], [10]. Within this time frame, microorganisms can sense and respond (or not) to drivers and change their distribution rapidly, driven by a variety of physical and (bio)chemical stimuli, such as light, electric fields, and nutrient availability [8]. The “instantaneous” distribution and growth of each microbial species are determined according to its ability to respond to drivers and successfully colonize nutrient-rich hotspots or plant hosts, in the case of pathogens and symbionts. Thus, the ability to capture phenotypic changes in real time in multiple species simultaneously at this granular time scale offers the possibility of characterizing key drivers and establishing additional rules of microbiota assembly.

Microfluidics offers the opportunity to design geometrically defined microdevices targeted for the in situ or “instantaneous” analysis of low volumes of microbial samples [11]. Such an approach can provide phenotypic information on time scales of seconds to minutes which is fundamental. Integrating specially designed, controlled microfluidic circuits with automated cell tracking and image analysis has already enabled the quantification of microbial dynamics at high spatial and temporal resolutions. Microscopy-based studies of fluorescently labelled bacterial species in environments around plant roots has contributed to the understanding of plant-pathogen-beneficial species interactions [12]. Similarly, image-based analysis of confined pathogenic zoospores in a microfluidic set-up made it possible to precisely quantify the motion in response to a soil driver [13].

At the same time, advances in automated cell tracking have also recently been made with the introduction of deep learning-based object tracking, resulting in improved performance, usability, and versatility [14], [15], [16]. Deep-learning algorithms have been employed for object tracking in microdevices, enabling precise quantification of the responses of individual microbial swimming species to specific environmental drivers. These microfluidic platforms, featuring diverse geometric configurations, have shed light on the navigational strategies (including direction, trajectories, speed) of organisms such as the dinoflagellate *Oxyrrhis marina* in chemoattractant gradients, the green algae *Chlamydomonas reinhardtii* in varying light conditions, and the effects of KCl on *Pyramimonas octopus* [17], [18]. The advent of multilayer microfluidic technologies further expands the scope of ecological observations, allowing for the co-culturing of multiple species [17]. A challenge now is to integrate these tools to characterize the effect of a driver, not only on cells of a single species but simultaneously on multiple species representative of a microbiota or a synthetic microbial consortium [19].

Here, we couple microfluidic technologies with an automated cell tracking program in order to investigate the behavioral response of three different telluric species within a synthetic community to a spreading driver, a controlled potassium gradient. In our case, we evaluated the chemotactic effect of the potassium gradient on *Phytophthora parasitica* zoospores, *Vorticella microstoma* protozoa cells and *Enterobacter aerogenes*. *Phytophthora parasitica* is a filamentous eukaryotic plant pathogen that causes disease in natural and agricultural systems worldwide, spreading in water films by bi-flagellate zoospores known to respond to potassium gradients with negative chemotaxis [13], [20]. *Vorticella microstoma* is a suspension-feeding ciliate living in freshwater and soil habitats, in two forms: free-swimming telotrochs and sessile stalked trophonts [21]. Sessile stalked trophonts are suspension-feeding forms, using oral cilia beats to generate a water vortex flow to draw bacteria food particles toward a mouth-like part, the peristome [21], [22]. *Enterobacter aerogenes* are motile gram-negative bacteria, associated with a variety of environmental habitats [23], including soil [24], where they can act as endophytic plant bacteria [25].

By coupling the use of microdevices to control potassium delivery [13] and the open source software TrackMate for cell tracking [16], we have developed a method that enables simultaneous, automated, image-based characterization of the behavior of three distinct microbial species and their distributions in response to a driver. We applied morphometric analyses and performed single cell tracking to identify and characterize each cell of the three species in the suspension and then discriminate their specific environmental responses in terms of motion dynamics. This method (i) enabled species-specific characterization of the micro-behavior and micro-distribution of cells in a mixed synthetic suspension when exposed to a driver, at high temporal and spatial resolution; and (ii) provided the capability to identify additional influencing factors, such as intraspecific interactions. Our approach demonstrates that the integration of microdevices and videomicroscopy can significantly contribute to our understanding of microbial dynamics and diversity at microscales, as well as elucidating how various drivers shape microbiota assemble at the micrometric level. Such advancements may contribute to microscale investigations which are especially challenging in microbial ecology [19], [26], [27].

## 2 Methods

### 2.1 Oomycete, ciliate and bacterial strains

The choice of using the strains *Phytophthora parasitica*, *Vorticella microstoma* and *Enterobacter aerogenes* was determined by different factors. The different cell sizes of *P. parasitica* zoospores,*V. microstoma* ciliates and *E. aerogenes* bacteria (diameters greater than 10, 30 and 2 µ*m*, respectively) were suitable to easily detect each species and monitor their distribution under the microscope. Furthermore, the well-known motion response of *Phytophthora* zoospores to a potassium gradient made it possible to use their motion properties to verify the automated cell tracking analysis. The *Phytophthora parasitica* (isolate 310) strain was obtained from the *Phytophthora* INRAE collection (Sophia Antipolis). The *Vorticella microstoma* 30897 and *Enterobacter aerogenes* 13048 strains were purchased from the American Type Culture Collection (ATCC).

### 2.2 Culture conditions and synthetic community cell suspension

*Phytophthora parasitica* was cultured on malt agar at 24^◦^C. Mycelia were cultured for one week in V8 liquid medium at 24^◦^C under continuous light. The material was then dilacerated and incubated for a further four days on 2% agar in water. The zoospores were released as described by Larousse et al. [28]. *Vorticella microstoma* and *Enterobacter aerogenes* cells were cultivated for 3–4 days in V8 liquid medium at 24^◦^C with a 16-h photoperiod. Ciliate and bacterial cells suspended in liquid medium were separated from the flocculates through a 15 min decantation step of 10 *mL* of the culture and recovery of the upper 0.5 *mL*. The mixed suspensions of *P. parasitica* (P), *V. microstoma* (V) and *E. aerogenes* (E) cells were generated in water and calibrated at about 200, 10 and 2000 cells µ*l*, respectively.

### 2.3 Set-up used for cell motion analysis

Each community cell suspension (50 µ*l*) was gently mixed before being placed in a commercial Ibidi microchamber (µ-Slide VI—flat; Ibidi; size l: 17 mm; w- 3.8 mm; h: 400 µ*m*). A passive dispersion system was used to generate an environmental gradient of potassium, and to create a flowfree and shear-free environment for cell sensing. The diffusion-based potassium gradient was generated by adding 0.5 µ*l* of 500 mM KCl to a lateral open inlet as described in Galiana et al. [13]. At the 5 min time point, cell motion was captured with an Axio Imager Z1 microscope (Zeiss) equipped for bright-field microscopy. The Axiovision 4.6 (Zeiss) software was used for the acquisition of movies, generating sequences of 10 s at a frame interval of 0.07350 s (Appendix A: Supplementary File 1; Supplementary File 2; Supplementary File 3).

### 2.4 Image treatment

Image pre-treatment and analysis were performed with the open-source Fiji software package [14]. To facilitate the comprehension of the manuscript when referring to the microbial cells, we use the term spots or objects, in line with Fiji language. Three different movies corresponding to three different biological replicates were first converted into an 8-bit color graphic and reported with the correct size scale. Each movie was then transformed into a mask of binary images where regions of interest (ROIs) corresponding to biological objects were converted into black spots (pixel value=255) over a white background (pixel value=0). Considering that our mixed suspension was made of different-sized species, threshold values below 100 were insufficient to include smaller-sized objects (here bacteria and zoospores cells) that were defined by a smaller number of pixels. On the other hand, values greater than 118 risked grouping together pixels representative of background areas where light was overexposed. Based on these considerations, to obtain final binary images in the conditions used, we applied threshold values between 100 and 118. We then performed the analysis of cell motion dynamics, as described below, at millimeter and micrometer scales. In the latter case, to better define the micrometric areas to analyze, we created different reference grids for each replicate, composed of squares with sides of approximately 170 µ*m*. The analyses using this grid size enabled us to define six different microenvironment areas as detailed below in the results section.

### 2.5 Automated cell-image analysis

Morphometric and motion dynamics data were generated as follows and synthesized in a step-by-step protocol workflow (Appendix A: Supplementary File 4). Dynamics of cell motion were investigated using TrackMate version 7, incorporating algorithms for display and quantifying the shape of objects in 2D and 3D [16]. In the TrackMate environment, objects are analyzed by two operational modules, one which allows the detection and filtering of the objects, or spots, present and a second one in which a tracker module links together the filtered spots to build tracks. Here, to detect and filter spots of interest in the previously obtained binary masks, we selected the 2D mask detector option, which results in the individuation of all spots by delineating each different perimeter and calculating areas or shape scores. The mask detector creates objects from a black and white channel in the 8-bit source image, based on the pixels having values strictly larger than 0. To include as many of the smaller bacteria objects as possible, we deselected the option of simplifying the contour of spots from the detector panel. For the majority of bacterial cells, this option was not able to resolve the already small number of micrometer-sized bacteria shapes into smoother ones characterized by fewer segments. Following the selection with the mask detector and to further allow each biological object to be distinguished and analyzed separately, we set the spot detection parameters. Initial threshold quality was set at 1 in all three cases. This initial spot thresholding is based on the quality value set by the segmenter to indicate the likelihood that each spot is a relevant detection. This value can be increased if it is believed that the quality of the identified spots is not sufficient (if for example it might include noise particles from background or general spurious spots), or if the number of detected spots is very large (https://imagej.net/plugins/trackmate/tutorials/getting-started).

Next, the radius of spots (accessible by setting filters on spots) was set with the following thresholds: above values between 10 and 13.5 µ*m* for *Vorticella* and below values between 2 and 4.5 µ*m* for *Enterobacteria*. In the case of *Phytophthora* zoospores, we set the radius filter twice on the spots. In the first filtering we selected spots with a radius below 10–12 µ*m*, in order to exclude *Vorticella* objects; in the second we selected spots with a radius above 4–5 µ*m*, to distinguish zoospores from bacteria cells. Measurement of the radius for each microbial cell was easily and quickly performed before the TrackMate analysis on the transformed binary images, by selecting the “straight” line icon on the panel and “measure” option in the “analyze” menu.

Tracking of trajectories was performed using the Nearest-neighbor tracker algorithm, which links spots between consecutive frames, by minimizing the global displacement of the particles. In the tracking settings, we tested different maximal linking distance values that made it possible to visually distinguish the trajectories of the three types of cells. We finally adopted maximal linking values of 15 µ*m* for P, 60 µ*m* for V, and 2 µ*m* for E, knowing that the fastest expected species was *V. microstoma* and the slowest was *E. aerogenes*. The maximal linking distance for the expected speed of each microbial species was also estimated with the manual tracking option in ImageJ (https://imagej.nih.gov/ij/plugins/track/track.html). From the resulting analysis tables automatically generated by TrackMate, we extrapolated and plotted estimations of the areas and perimeters of spots associated with each trajectory, to identify the three species by their morphologies, and to calculate the mean speed of cells, in order to analyze their specific behavioral response. In the particular case of cell motion analysis at the micrometer scale, we determined mean speed and confinement ratio (persistence) measurements. The confinement ratio is defined as the ratio between the net displacement and the total distance travelled by each spot, that is, by each microbial cell [29]. Hence, values close to 0 indicate a confined movement, where microbial particles remain close to the starting point of their path or displacement. Values close to 1 indicate that microbial particles are moving away from the starting point of the track with constant orientation. Statistical analysis was performed using a one-way analysis of variance (ANOVA) test with a Sidak post hoc test for multiple comparisons (GraphPad Prism version 10, GraphPad Software, Boston, Massachusetts USA).

## 3 Results

### 3.1 Morphological discrimination of the three species

Detection of spots through the mask detector of TrackMate enabled us to discriminate between the three different groups of spots using the expected areas and perimeters of the three microbial cells.

**Fig. 1:**
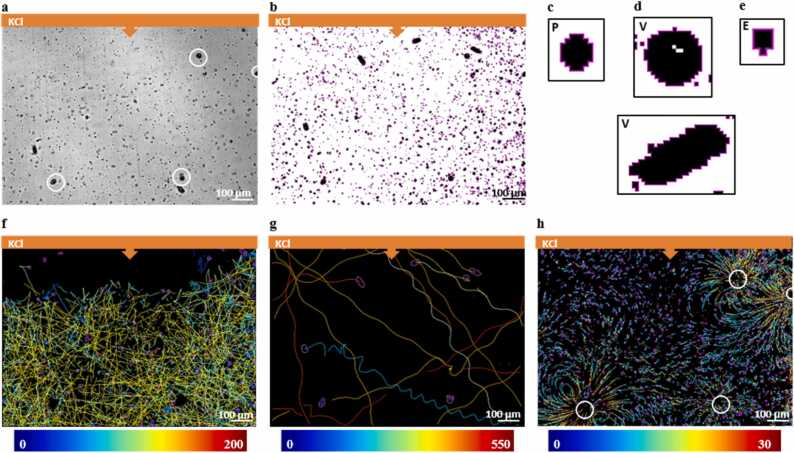
Partition of spot shapes, trajectory patterns of the three different microbial species upon imposition of the potassium gradient. (1a) A representative overview of the original frames. The potassium solution (KCl) diffused passively from the inclusion point indicated by the orange bar on the top of the image. (1b) The mask detector delineated the shapes of the three different morphological spots on the transformed binary frames and partitioned them based on the defined TrackMate parameters. (1c) The shape of spots associated with *P. parasitica* zoospores (P). (1d) The circular shapes associated with sessile forms of *V. microstoma* (V) on the top; ellipsoidal shapes associated with free-swimming forms of *V. microstoma* (V) on the bottom. (1e) The detected spot shapes associated with *E. aerogenes* cells (E). (1f-g-h) trajectory patterns and mean velocity ranges generated after partition of the 3 groups of spots (1f). The confined trajectory pattern of *P. parasitica* zoospores (group 1) displayed negative chemotactic behavior in response to the potassium gradient, swimming away from higher concentrations. (1 g) *V. microstoma* (group 2) exhibited mostly linear or sinusoidal trajectories. Sessile forms, encircled in white in 1a and 1 h, did not display any trajectory. (1 h) Trajectories of *E. aerogenes* (group 3) in proximity to sessile *V. microstoma* showed characteristic patterns of vortex flows around the sessile *Vorticella*. At the bottom of the trajectory images 1f, 1 g, and 1 h is the corresponding mean speed (µ*m/s*) color bar, varying from blue/green (low speed) to yellow/red (high speed). Movies were obtained at 10x magnification and analyzed in the Fiji environment. Scale bars: 100 µ*m*.

**Fig. 2:**
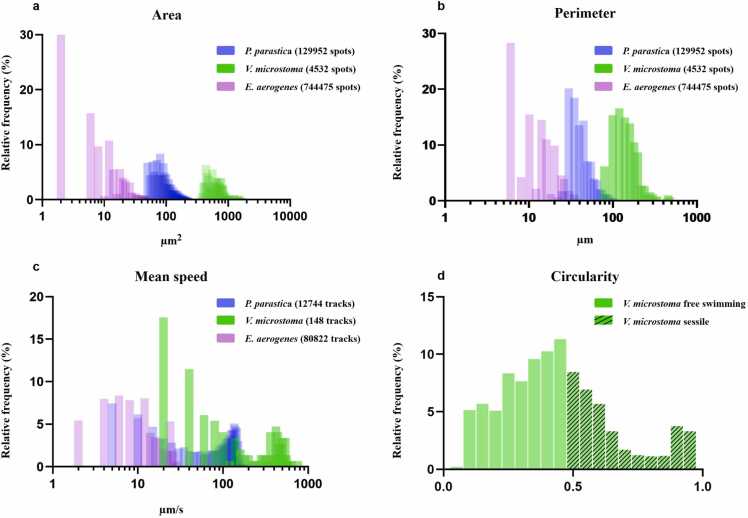
Frequency distributions of morphometrics and motion characterizing the three partitioned groups. (2a-b-c) Frequency distributions of the areas (2a), perimeters (2b) and mean speeds (2c) of the three grouped species based on data merged from the three replicates. As expected, the order from the smallest to the largest species in the suspension was *V. microstoma*, *P. parasitica* and then *E. aerogenes*. The y axis shows the relative frequency as a percentage (%); the x axis shows the area, perimeter, and mean speed measurements (µ*m*^2^, µ*m* and µ*m/s*) on a log10 scale. (2d) The frequency distribution of circularity values for *V. microstoma* cells (data merged from the three replicates). Values between 0.5 and 1 indicate the more circular *V. microstoma* sessile forms. The y-axis shows the relative frequency as a percentage (%); the x axis shows the circularity measure.

The total number of spots for each group detected over the three movies (a total of 430 frames; Table 1) was 129 952 spots for group 1, which corresponds to *P. parasitica* zoospores(Fig. 1c); 4532 spots for group 2, which corresponds to *V. microstoma* cells (Fig. 1d) that exhibited either smaller and circular shapes or larger and ellipsoidal shapes, in their sessile or free swimming states, respectively; and 744 475 spots for group 3, which corresponds to the smallest detected spots, associated with *E. aerogenes* cells Fig. 1e). Group 1 exhibited area and perimeter values between 50 and 200 µ*m*^2^ and 30–100 µ*m*, respectively (Figs. 2a-2b). Group 2 presented the highest area and perimeter values, with the majority of the spots having area and perimeter values ranging between 400 and 1000 µ*m*^2^ and 100–200 µ*m*, respectively (Figs. 2a-2b). Circularity parameters retrieved from TrackMate further confirmed the double-shape status of *V. microstoma*. The majority of protozoa cells had circularity values between 0 and 0.5, likely to represent its ellipsoidal-like free-swimming forms, while a minority showed circularity values close to 1, corresponding to the more circular sessile forms of the ciliate (Fig. 2d). In group 3, 60% of the detected spots had an area of approximately 12 µ*m*^2^ as maximum and almost 75% of spots exhibited perimeter values between 0 and 15 µ*m* (Figs. 2a-2b).

**Table 1:** Analysis of replicates for morphometric and motion observations. For each replicate, the three microbial species presented different numbers of spots and tracks detected, and different calculated uniform mean values of the considered parameters (area, perimeter, mean speed). *V. microstoma* presented a limited number of cells and tracks.

Replicate	Microbial Species	Number of spots	Number of Tracks	Area *µm*^2^ [min-max]	Perimeter *µm* [min-max]	Mean speed *µm/s* [min-max]
Replicate 1	*P. parasitica*	26966	2918	[79–360]	[37–198]	[0–213]
	*V. microstoma*	973	32	[451-1500]	[101-256]	[2-540]
	*E. aerogenes*	326387	31004	[2–56]	[6–67]	[0–28]

Replicate 2	*P. parasitica*	44713	4189	[48–340]	[26–141]	[0–210]
	*V. microstoma*	2271	73	[354-2485]	[84-169]	[1-760]
	*E. aerogenes*	275901	24843	[2–25]	[6–33]	[0–28]

Replicate 3	*P. parasitica*	58273	5637	[46–239]	[25–166]	[0–211]
	*V. microstoma*	1288	43	[397-1892]	[73-335]	[2-648]
	*E. aerogenes*	142187	24975	[2–32]	[8–43]	[0–28]

### 3.2 Distribution and behavioral response of the microbial species to a potassium gradient at the millimeter scale

A passive dispersion system generated by adding a potassium solution to the lateral open inlet of the microchamber was used to investigate the motion responses of the three different microbial groups. The point of potassium inclusion, which corresponds to the area that maintained the maximum concentration of the ion throughout the observation, corresponded to the upper side of the resulting images (Fig. 1a).

To describe the motion dynamics adopted by the microbial species after 5 min of potassium diffusion, we first determined the trajectories found for each species (Figs. 1f-1g-1h). Short trajectories indicate spots (microbial cells) that travelled short distances or parts of long trajectories not fully tracked by the TrackMate Fiji plug-in. Non-motile spots are mainly observed within their profile shapes and do not display any trajectory pattern (Figs. 1f-1g-1h). As expected, P. parasitica zoospores (12 744 tracks) occupied the millimetric space heterogeneously, displaying the characteristic negative chemotaxis in response to high concentrations of potassium (Fig. 1a) [13].

The net linear trajectories were mostly found in the area below the potassium inclusion point, while only a few nearly immobile forms (null or short trajectories) were detected in its vicinity (Fig. 1f). In contrast, protozoa cell trajectories appeared uniformly distributed with a linear pattern along the entire millimetric environment analyzed, without exhibiting significant spatial preference in relation to the KCl gradient (Fig. 1g). Finally, bacterial cells (80 822 tracks), mainly displayed two different types of trajectories, a curved and circular-like trajectory in the proximity of *V. microstoma* sessile forms, and a more linear and shorter trajectory in the surrounding areas (Fig. 1h). The bivalent bacteria displacement pattern occupied the entire millimetric space, regardless of the potassium concentration.

Using the parameters defined for the three trajectory patterns, we estimated the associated mean speed values for each microbial group (Fig. 2c). The mean speed distribution of the zoospores displayed a bimodal pattern, with a minority of cells having a mean speed of 4–20 µ*m/s* and a majority having mean speed values of 100–200 µ*m/s* (Fig. 2c). *Phytophthora parasitica* zoospores with null trajectories and very low mean speed values (below 10 µ*m/s*) were considered as effectively non-motile (Fig. 1f) (Fig. 2c). Similarly, over a total of 148 tracks, *V. microstoma* cells displayed mean speed values distributed mainly over two intervals, with approximately 30% of spots having mean speeds of 20–60 µ*m/s* and the rest having speeds between 80800 µ*m/s* (Fig. 2c). *Vorticella microstoma* cells with null trajectories and mean speeds below 20 µ*m/s* were considered as corresponding to the sessile forms of the protozoa, while all others with higher mean speeds and longer trajectories were considered, and easily characterized in the movies as, the free-swimming telotroch forms of the protozoa (Fig. 1g) (Fig. 2c). The substantial fraction of bacterial cells (*<* 40%) moved at a very low mean speeds, below 20 µ*m/s* (Fig. 2c).

### 3.3 Analysis of motion and behavior at the micrometer scale revealed other environmental interactions

**Fig. 3:**
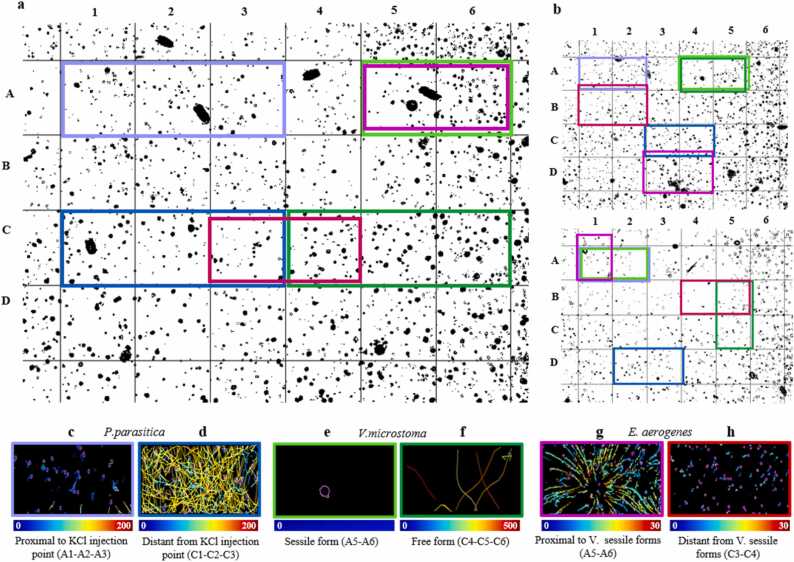
Definition of six different microenvironments based on trajectory patterns. (3a) Example of the grid applied to replicate 1. The grid cells corresponding to each microenvironment are outlined in different colors. The colors are labelled in 3c-h. (3b) The grids applied to the other two movie replicates and the respective colored labels. (3c,d,e,f,g,h) Trajectory patterns for six different microenvironments in replicate 1: from left to right *P. parasitica* (proximal to KCl injection point vs. distant from KCl injection point) from cells A1-A2-A3 and C1-C2-C3 of the grid; *V. microstoma* analysis (sessile vs free-swimming forms) from cells A5-A6 and C4-C5-C6; *E. aerogenes* analysis (in proximity to *V. microstoma* (V) sessile forms vs. distant from V sessile forms) from cells A5-A6 and C3-C4. The color bar below each image indicates the mean speeds (µ*m/s*).

**Fig. 4:**
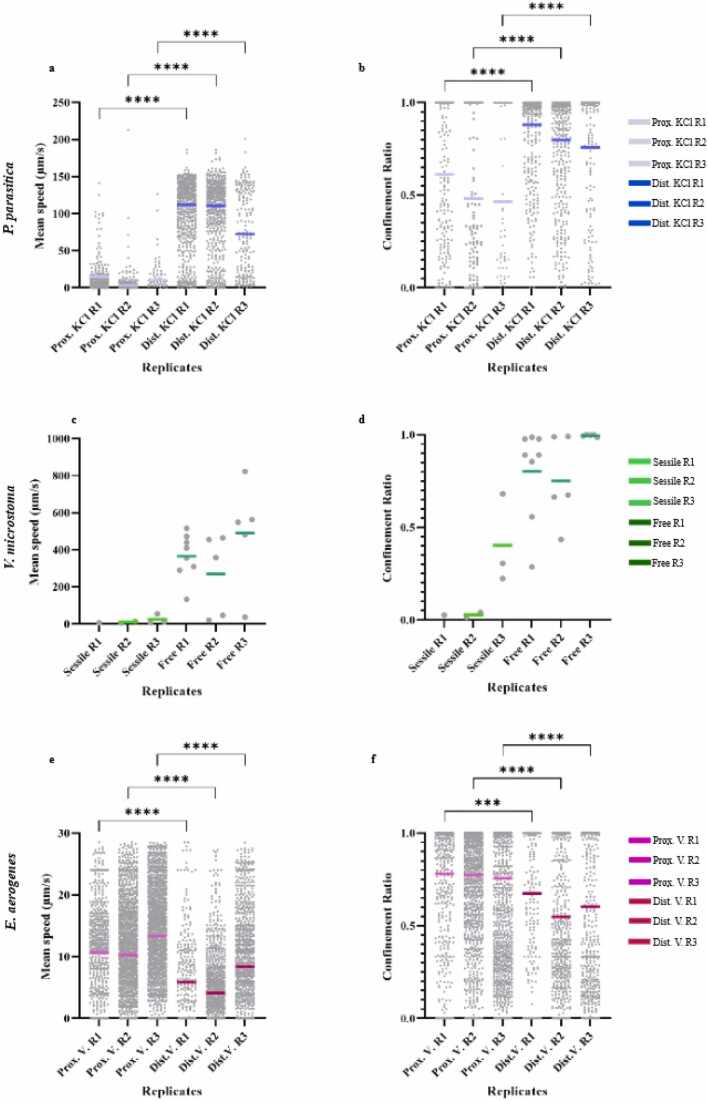
Analysis of mean speed and confinement ratios in the six different defined microenvironments. (4a; 4b) *P. parasitica* zoospores proximal to or distant from the KCl injection point (total number of tracks: 453 and 1164, respectively). (4c;4d) *V. microstoma* free-swimming forms (total number of tracks: 18) vs *V. microstoma* sessile forms (total number of tracks: 6). (4e;4f) *E. aerogenes* proximal to or distant from *V. microstoma* (sessile forms) (total number of tracks: 4222 and 2588, respectively). *** and **** indicate a significant difference calculated by a one-way analysis of variance (ANOVA) test with a Sidak post hoc test between the two conditions considered in each replicate where (*p* = 0*.*0007) and (*p* < 0*.*0001).

To further analyze the properties of the synthetic microbial community suspension, a systematic screening of the movies at the micrometer scale was performed using a grid with squares of unit area 170 × 170 µ*m*^2^, corresponding to a coordinate system of letters and numbers (Figs. 3a-3b). At this resolution, the study allowed us to identify two distinct motion behaviors for each species, occurring in particular microenvironment areas. In the most obvious cases of *P. parasitica* and *E. aerogenes*, we were able to discriminate and further investigate their motion behaviors in two microenvironments where *P. parasitica* was subjected, or not, to a KCl gradient, and *E. aerogenes* was in proximity, or not, to the *V. microstoma* sessile forms. For *V. microstoma*, we deliberately defined a microenvironment characterized by the presence of *V. microstoma* sessile forms and one characterized by *V. microstoma* free-swimming forms. As a result, for each replicate, six different microenvironments were established (Table 2) (Figs. 3c-3d-3e-3f-3g-3h). To normalize our observations, we selected for each microenvironment approximately the same number of grid cells for each species in each replicate. For each species, we analyzed the same number of frames (Table 2), within the same replicate. The frames selected were based on the best spatial and temporal placement, so that no interference with the other microorganisms in the suspension or in other microenvironments occurred. For each microenvironment, we analyzed the mean speed and confinement ratio values of the three species in order to describe their distinct motion dynamics (Fig. 4).

**Table 2:** Replicates and corresponding microenvironment analyzed for the three microbial species. Microenvironments were identified using the grid cells illustrated in Fig. 3. For each replicate and microenvironment, the three microbial species presented different numbers of spots and tracks detected. (*) in *E. aerogenes* microenvironment (Proximal to V) of Replicate 2 indicates that the considered grid cells include part of the underlying E3 and E4 cells; (*) in *E. aerogenes* microenvironment (Proximal to V) of Replicate 3 indicates that the considered grid cells include part of the above A1 cell.

Replicate	Microbial species	Microenvironment	Frames	Grid cells analyzed	Number of Spots	Number of Tracks
Replicate 1	*P. parasitica*	Proximal to KCI	[1–143]	A1-A2-A3	4248	231
		Distant from KCI	[1–143]	C1-C2-C3	3839	505
	*V. microstoma*	Sessile forms	[1–114]	A5-A6	114	1
		Free forms	[1–114]	C4-C5-C6	52	8
	*E. aerogenes*	Proximal to V	[1–81]	A5-A6	3626	661
		Distant from V	[1–81]	C3-C4	2234	332

Replicate 2	*P. parasitica*	Proximal to KCI	[1–143]	A1-A2	2758	166
		Distant from KCI	[1–143]	C3-C4	5123	480
	*V. microstoma*	Sessile forms	[1–43]	A4-A5	86	2
		Free forms	[1–43]	A4-A5	47	5
	*E. aerogenes*	Proximal to V	[1–143]	D3-D4*	10209	1580
		Distant from V	[1–143]	B1-B2	11742	1060

Replicate 3	*P. parasitica*	Proximal to KCI	[1–41]	A1-A2	552	56
		Distant from KCI	[1–41]	D2-D3	1044	179
	*V. microstoma*	Sessile forms	[1–25]	A1-A2	51	3
		Free forms	[1–25]	B5-C5	16	5
	*E. aerogenes*	Proximal to V	[1–143]	A1*	8727	1981
		Distant from V	[1–143]	B4-B5	6316	1196

Microenvironment analysis revealed that, for each replicate, *P. parasitica* zoospores showed two different sets of mean speed values (ANOVA test, *p* < 0*.*0001) (Fig. 4a) depending on the proximity (Fig. 3c), or not (Fig. 3d), to the potassium injection point. In the microenvironment where the zoospores exhibited negative potassium chemotaxis, moving away from the potassium injection point, the mean speed value for the three replicates was higher, with values above 50 µ*m/s* (Fig. 4a). In replicates 1 and 2, most zoospores reached a velocity of 150 µ*m/s*, while fewer zoospores belonging to replicate 3 reached the same value (Fig. 4a), suggesting the presence of a higher number of nonmotile zoospores. Conversely, mean speed values dropped to 0 µ*m/s* when zoospores were moving in the microenvironment corresponding to highest potassium concentrations (micro area around the injection point) (Fig. 4a). Non-motile zoospores exhibiting low mean speed values corresponded to their deflagellated and encysted forms previously described in the literature in the presence of a high concentration of potassium [20].

Mean speed analysis of the two microenvironments associated with *V. microstoma* revealed values mostly above 400 µ*m/s* (Fig. 4c) for long trajectories of free-swimming forms of protozoa and approximately between 0 and 50 µ*m/s* (Fig. 4c) for sessile forms (Figs. 3d-3e) independently of their proximity to the injection point of KCl. Subaverage mean speeds (spanning down to 0 µ*m/s*) found in the microenvironments of the free-swimming forms of *V. microstoma* in replicates 2 and 3 (Fig. 4c), are explained by the colocalization of a sessile form of the same species within the considered frames. Considering the limitations of ANOVA statistical tests for small sample sizes, we did not perform any statistical tests for the *V. microstoma* cells group, which had only 24 tracks in total (Table 2).

The investigation of the two microenvironments associated with different *E. aerogenes* behavior revealed two sets of mean speed values: one related to bacteria located far away from *V. microstoma* sessile forms (Fig. 3h) with mean speed values on average below 10 µ*m/s* (Fig. 4e), and a second related to bacteria located close to *V. microstoma* sessile forms with a higher mean speed (on average above 10 µ*m/s*) (Fig. 3g) (Fig. 4e). The results were further confirmed by the ANOVA statistical test, showing the difference between the two conditions tested (*p* < 0*.*0001). In line with millimetric observations (Fig. 1h), we again observed more circular trajectories of bacteria near and around the sessile protozoa forms, and shorter and straight trajectories for bacteria more distant from sessile protozoa forms. Together, these circular bacterial trajectories formed a circular vortex due to the water flow dragging force generated by the oral cilia beating of *V. microstoma* sessile trophont forms [21].

For each microenvironment and estimated cell tracks, we also analyzed confinement ratio rates. Further ANOVA statistical tests were performed only for the *P. parasitica* and *E. aerogenes* groups. As expected, values mostly distributed closer to 0 were recorded for *P. parasitica* zoospores close to the potassium injection point (Fig. 4b) and immobile forms of *V. microstoma* (Fig. 4d), indicating a more constrained movement within the micro area. In contrast, a broader range of confinement ratio values, closer to one, were recorded for *P. parasitica* zoospores farther from the potassium injection point (Fig. 4b) and for *V. microstoma* motile forms (Fig. 4d), representing a larger displacement along the trajectories drawn. Similarly, although with slightly less significance, at least for replicate 1 (*p* = 0*.*0007), *E. aerogenes* showed larger displacement for cells impacted by *V. microstoma* sessile water flow and more constrained movement for cells far from sessile protozoa cells (Fig. 4f).

## 4 Discussion

Prokaryotic and eukaryotic microorganisms possess the ability to respond to various environmental clues. Detection and response to exogenous signals results in morphological, physiological, growth and motility changes [30], [31]. In soil, multiple biotic and abiotic factors affect the biology of microorganisms and thus mediate microbiota assembly [1], [32]. Determining these drivers is a key issue in soil microbial ecology. In studies of drivers, the understanding of their impact at the spatio-temporal level remains rudimentary. Most of the current information related to these two topics are mainly based on metagenomic sequencing of environmental samples or on the use of synthetic microbial communities to assess functional microbial diversity and abundance of microorganisms subjected to environmental factors [4].

In this study, we showed that phenotypic data can be generated simultaneously for multiple microbial species using microfabrication coupled with automated tracking. We quantify microbial morphologies and trajectories, illustrating real-time kinetics of microbial distribution, assembly, and dispersion upon driver application. Such an approach made it possible to, firstly, accurately partition and morphologically characterize three cell species (*P. parasitica*, *V. microstoma* and *E. aerogenes*) that form a simple synthetic community, and secondly, to determine their specific motion response to the selected soil driver (potassium), and reveal further secondary interactions.

Morphological differences among the three different species were identified using the TrackMate mask detector. Three categories of spots corresponding to the three microbial cells were differentiated according to size criteria (cell radius) and separately analyzed to retrieve morphometric parameters (area and perimeter). The three size-partitioned groups were then independently analyzed by the Nearest-neighbor tracker to determine cell-specific motion and behavior characteristics. Consistent with previous findings, this partition makes it possible to easily extract and distinguish the expected behavior of the three species. We indeed verified the negative chemotactic behavior adopted by *P. parasitica*, which moved towards areas with lower potassium concentrations and encysted, after de-flagellation when unable to escape areas with higher potassium concentrations [13], [20]. Similarly, we observed the dual biological status of *V. microstoma*, that, independently of the potassium gradient, pervaded the microbial community both with its sessile and free-swimming forms. Most notably, we observed interesting intra-species interactions between *V. microstoma* and *E. aerogenes*, characterized by water flow patterns generated by *Vorticella*’s oral cilia that drew in nearby bacteria particles as source of nourishment. Interestingly, our results showed that even bacteria distant from *Vorticella* exhibited movement, albeit with reduced motion metrics (Fig. 3c). Considering that a single *Vorticella* can influence particles up to 450 µ*m* away [21], we assumed that in our 1000*800 µ*m* environment, these distant bacteria were still affected by *Vorticella*’s generated water flow, albeit less dramatically. This assumption was supported by observations of bacterial dynamics in isolated conditions, where the absence of *Vorticella* led to either non-motile or confined bacterial movements with shorter and non-linear trajectories, likely due to Brownian motion (Appendix A: Supplementary File 5).

Thus, the results achieved so far demonstrate the efficiency of the proposed approach that offers the ability to characterize the behaviors of at least three species in a mixed suspension and their displacements in the milli- or micrometric environment considered. Of course, to go beyond n = 3 as in this study, more work is needed to understand how a particular driver affects the complex assembly of a microbiota. However, we conjecture that it should be feasible to investigate rules of assembly for about ten species at the same time, considering that, in one dimension, the cells could exhibit specific morphological traits and, in a second dimension, cells should express fluorescent proteins with minimal spectral overlap [33]. Thus a method could be developed for a mixed-species sample, using the approach described in this study for the first dimension, and capturing the second dimension by measuring the fluorescent signals in successive sequences of the same movie using the optical filters conventionally used in fluorescence microscopy. Tracking could be carried out by exploring the new segmentation tools introduced in the current version of TrackMate [16], which enable single-frame tracking of the shape and position of the different fluorescent objects. Developing such approaches might enable the investigation of both phenotypic and genetic data on co-cultured multiple and representative taxa, thereby contributing to the understanding of the assembly, structure, and functional dynamics of the microbiome [34].

Other critical points of the approach must be addressed to develop it further. For example, the image acquisition proved to be a crucial step, along with adopting the right thresholding values in the resulting output binary frames for further analyses. Since spot detection relies on discerning the pixels associated with the spots, maximizing the quality of the input video source (with no light variation) might enhance the quality and homogeneity of thresholding. In this way the distinction between smaller and larger microbial shapes, characterized by a smaller and larger number of pixels, respectively, would be facilitated and replicable in different conditions. However, as previously mentioned when the species considered are too morphologically similar, for example in area or perimeter, thresholding based on pixel values and sorting based on geometrical measurements are not enough for a good microbial partition. In this case other strategies, such the one above mentioned (partition based on fluorescence) might be adopted.

The approach described in this paper can be applied in the context of the evaluation of microorganisms as biocontrol agents for sustainable agriculture and precision crop management. The metrics based on the characterization and quantitative comparison of the behavior of a pathogen and biocontrol agents under various ecological drivers will provide information on co-distribution, assembly or dispersion of the pathogen and the microorganism under investigation. The ability of the microorganism to evolve and grow in the same microhabitat as the pathogen is an important additional parameter to have in hand to determine the effective use of a biocontrol agent for controlling the plant pathogen. Finally, as recent studies have proposed, such an approach might be also used to screen dynamic and real-time interactions between plant roots and different microbial species, in a root-on-a chip system [35], [12], [10]. This would enable the instantaneous observation of interaction dynamics and the definition of microbial hot spots of colonization relative to root-exudated areas.

## 5 Conclusion

We have developed an optimized, two-step approach for the efficient analysis of the response of a mixed synthetic microbial suspension to an external environmental driver. The first step is based on the morphological discrimination of the three species in suspension and the second step is to determine the species-specific displacement and distribution.

Based on the results, we can propose this as a valid and reproducible approach to characterize the dynamics of microbial communities and their interaction with their microenvironment at high-temporal scale. This method has the benefit of being simple to implement and cost-effective to replicate, as it relies on low-priced prefabricated microfluidic components and image analysis using open-source software. We can thus imagine applying this approach as a supplementary tool to determine properties of synthetic microbial communities within soil water films, or other habitats like fresh-water environments. In these cases, such an automated, tracking-based approach would enable the simultaneous analysis of the interactions of multiple microbial species. Similarly, in the field of plant disease management, the method might enable the screening of potential biocontrol agents, by determining the effect of drivers on co-distribution and interactions between pathogenic and beneficial microbial species and between biocontrol agents in the vicinity of a host plant.

## Funding

This work received support from the French government, managed by the French National Research Agency under the Investissements d’Avenir UCAJEDI project bearing the reference number ANR-15-IDEX-01. Carlotta A. Lupatelli received a PhD grant financed by the Graduate School LIFE, Graduate School SPECTRUM and Academy 4 (Université Côte-d’Azur). The work was also supported by the National Research Agency project number ANR22-CE20-0021 and by the INRAE Plant Health and Environment Division through the “Action Recherche”.

## CRediT authorship contribution statement

Conceptualization: CL, EG, AA, XN; Formal analysis: CL, EG, MK; Funding acquisition: CL, EG, AA, XN; Methodology: CL, EG, PT; Project 503 administration: EG; Supervision: EG, XN, AA; Validation: EG, XN, AA, 504 CC, PT; Visualization: CL; Writing - original draft: CL and EG; Writing 505 - review & editing: CL, EG, AA, XN, CC, PT. All authors have read and 506 agreed to the published version of the manuscript.

## Declaration of Competing Interest

The authors* declare that the research was conducted in the absence of any commercial or financial relationships that could be construed as a potential conflict of interest.
