# Supplementary material for: Automated high-content image-based characterization of microorganism behavioral diversity and distribution
Source: Comput Struct Biotechnol J. 2023 Nov 2;21:5640–9. doi: 10.1016/j.csbj.2023.10.055 (PMC10692603; doi:10.1016/j.csbj.2023.10.055)
Supplement: Supplementary file 1 — Supplementary material [file mmc1.docx]

**Appendix A.**

**Supplementary File 1** The original movie of microbial mixed-suspension (Replicate 1) is available in the link.

**Supplementary File 2** The original movie of microbial mixed-suspension (Replicate 2) is available in the link.

**Supplementary File 3** The original movie of microbial mixed-suspension (Replicate 3) is available in the link.

**Supplementary File 4** Method workflow illustration.


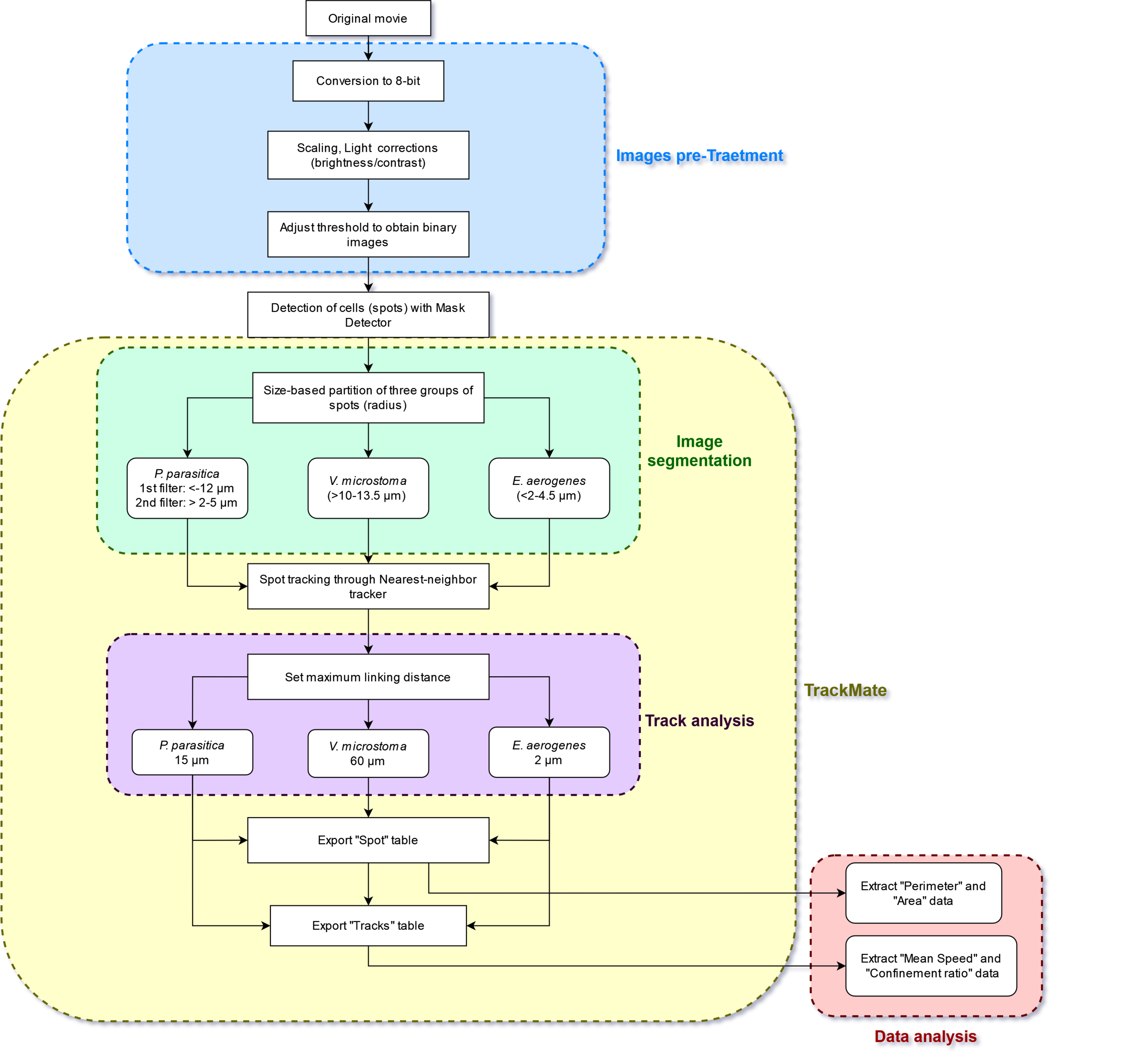


Figure A.5: Method workflow illustration. The workflow represents different steps of the image-based method for microbial mixed-suspension behavioral analysis.

**Supplementary File 5** Trajectories of isolated *E. aerogenes*.


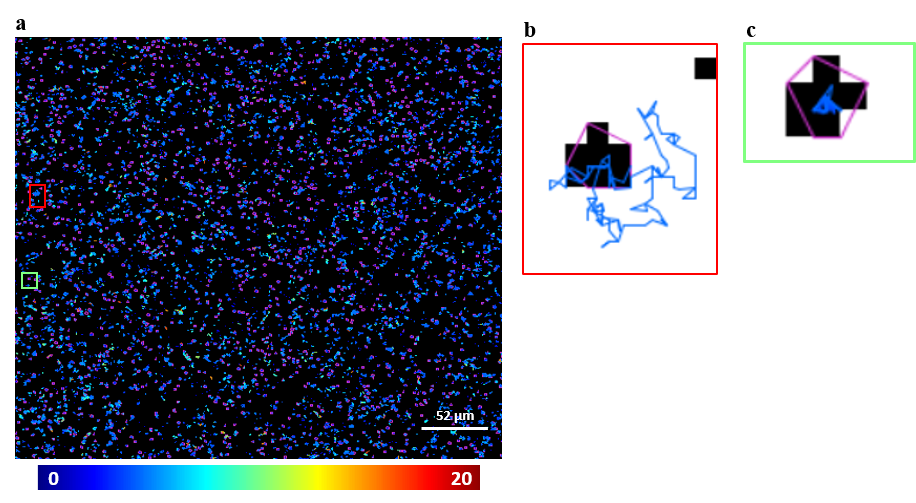


Figure A.6: Trajectories of isolated *E. aerogenes*. (a) An overview of isolated *E. aerogenes* in water suspension. Bacteria cells were isolated and diluted in water until reaching approximatively 2000 cells/*µl*. Bacteria cells were then confined in a microchamber in absence of potassium gradient. Isolated bacterial tracks (n=69314) displayed overall a confined movement with short and non-linear trajectories, probably consequential to the Brownian movement (b). A consistent part of *E. aerogenes* cells at this time point of the culture did not display any trajectory, therefore appearing to be non-motile (c). Only singular individuals appeared to be actively motile with longer trajectories characterized by higher mean speed recorded values (a). At the bottom of the image is the corresponding mean speed bar for tracked trajectories ranging from 0 to 20 *µm*/s. Three replicates were first obtained under 10x magnification, 0.044s frame interval and later analyzed under Fiji environment. Scale bar: 52 *µm*

**Supplementary File 6** Motion and morphological description of isolated *E. aerogenes*.


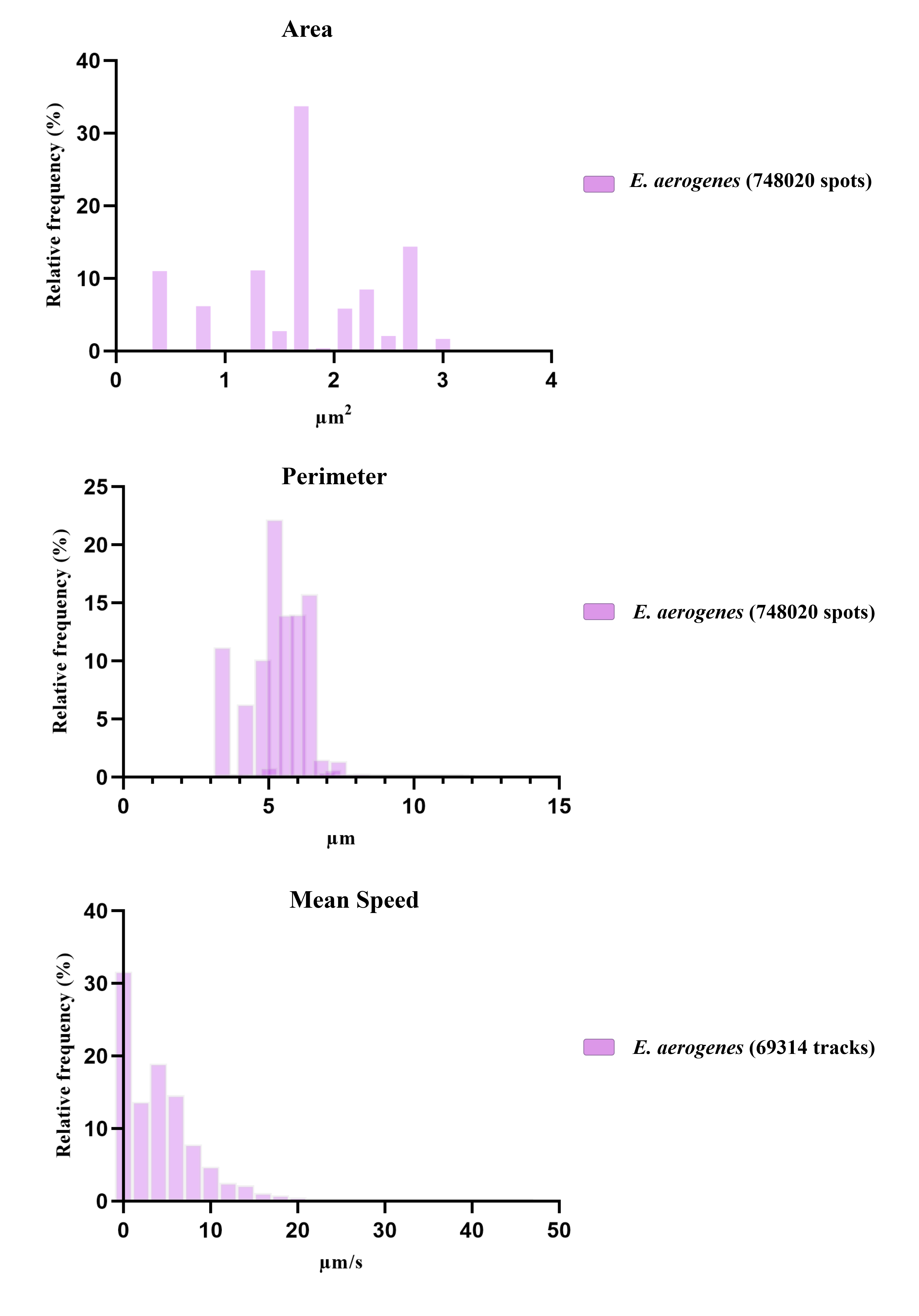


Figure A.7: Motion and morphological description of isolated *E. aerogenes*. Frequency distribution of morphometric and motion parameters (Area, Perimeter, Mean speed) characterizing isolated *E. aerogenes* bacteria. Over three different technical replicates (427 total frames), 748020 total spots were detected and associated to bacterial cells. In line with the analysis of bacteria population within mixed-suspension, majority of bacteria cells displayed area and perimeter values below 4 µm2 and 10 *µm*, respectively. Over 69314 tracks, around 90 % of bacteria displayed mean speed values below 10 *µm/s* . A 30% of *E. aerogenes* cells resulted to be non-motile with mean speed values equal to 0 *µm/s*. On the y axis the relative frequency in percentage (%), on the x axis the area, perimeter and mean speed measurements (*µm*^2^, *µm* and *µm/s*).
